# Course and Survival of COVID-19 Patients with Comorbidities in Relation to the Trace Element Status at Hospital Admission

**DOI:** 10.3390/nu13103304

**Published:** 2021-09-22

**Authors:** Gijs Du Laing, Mirko Petrovic, Carl Lachat, Marthe De Boevre, Georg J. Klingenberg, Qian Sun, Sarah De Saeger, Jozefien De Clercq, Louis Ide, Linos Vandekerckhove, Lutz Schomburg

**Affiliations:** 1 Laboratory of Analytical Chemistry and Applied Ecochemistry, Faculty of Bioscience Engineering, Ghent University, Coupure Links 653, 9000 Gent, Belgium; 2 Department of Internal Medicine and Paediatrics, Ghent University Hospital, C. Heymanslaan 10, 9000 Gent, Belgium; Mirko.Petrovic@UGent.be (M.P.); jozefien.declercq@ugent.be (J.D.C.); Linos.Vandekerckhove@UGent.be (L.V.); 3 Department of Food Technology, Safety and Health, Faculty of Bioscience Engineering, Ghent University, Coupure Links 653, 9000 Gent, Belgium; Carl.Lachat@UGent.be; 4 Centre of Excellence in Mycotoxicology and Public Health, Department of Bioanalysis, Faculty of Pharmaceutical Sciences, Ottergemsesteenweg 460, 9000 Gent, Belgium; Marthe.DeBoevre@UGent.be (M.D.B.); Sarah.DeSaeger@UGent.be (S.D.S.); 5 Institute of Experimental Endocrinology, Charité Universitätsmedizin, Hessische Straße 3-4, 10115 Berlin, Germany; g.j.klingenberg@me.com (G.J.K.); qian.sun@charite.de (Q.S.); Lutz.Schomburg@charite.de (L.S.); 6 Laboratory Medicine, AZ Jan Palfijn AV, Watersportlaan 5, 9000 Gent, Belgium; Louis.Ide@janpalfijngent.be

**Keywords:** micronutrient, nutrition, biomarker, diabetes, cancer

## Abstract

Selenium (Se) and zinc (Zn) are essential trace elements needed for appropriate immune system responses, cell signalling and anti-viral defence. A cross-sectional observational study was conducted at two hospitals in Ghent, Belgium, to investigate whether Se and/or Zn deficiency upon hospital admission correlates to disease severity and mortality risk in COVID-19 patients with or without co-morbidities. Trace element concentrations along with additional biomarkers were determined in serum or plasma and associated to disease severity and outcome. An insufficient Se and/or Zn status upon hospital admission was associated with a higher mortality rate and a more severe disease course in the entire study group, especially in the senior population. In comparison to healthy European adults, the patients displayed strongly depressed total Se (mean ± SD: 59.2 ± 20.6 vs. 84.4 ± 23.4 µg L^−1^) and SELENOP (mean ± SD: 2.2 ± 1.9 vs. 4.3 ± 1.0 mg L^−1^) concentrations at hospital admission. Particularly strong associations were observed for death risk of cancer, diabetes and chronic cardiac disease patients with low Se status, and of diabetes and obese patients with Zn deficiency. A composite biomarker based on serum or plasma Se, SELENOP and Zn at hospital admission proved to be a reliable tool to predict severe COVID-19 course and death, or mild disease course. We conclude that trace element assessment at hospital admission may contribute to a better stratification of patients with COVID-19 and other similar infectious diseases, support clinical care, therapeutic interventions and adjuvant supplementation needs, and may prove of particular relevance for patients with relevant comorbidities.

## 1. Introduction

The infectious coronavirus disease (COVID-19) caused by severe acute respiratory syndrome-coronavirus-2 (SARS-CoV-2) constitutes a life-threatening condition, in particular for a subgroup of patients with underlying comorbidities. Besides age [1], the most relevant risk factors for severe COVID-19 course and SARS-CoV-2 infection-associated death include hypertension, cancer, respiratory disease, obesity and diabetes mellitus [2,3]. These conditions are associated with metabolic dysregulation and inflammation [4], compromised signalling by reactive oxygen species (ROS) [5], and may involve a disturbed trace element status [6,7]. Among the important, intensively regulated and immune-relevant micronutrients are the essential trace elements copper (Cu), selenium (Se), and zinc (Zn) [8]. All three micronutrients are implicated in acute inflammation and in the concept of health-supporting nutrition to counteract ongoing inflammaging, i.e., the natural progressive age-dependent subclinical inflammatory processes finally causing organ dysfunction and degenerative diseases [9]. Accordingly, micronutrients may be of high relevance for reducing SARS-CoV-2 infection risk, supporting the immune system in combating the virus, and avoiding long-term adverse health issues from COVID-19 [10,11,12,13,14].

The micronutrient Se is needed for the biosynthesis of enzymatically active selenoproteins, including members of the families of glutathione peroxidases (GPX), thioredoxin reductases or iodothyronine deiodinases [15]. In view of their crucial roles in regulating reactive oxygen species (ROS) levels, energy metabolism and overall redox status in nearly all mammalian cells, Se status is closely interrelated with inflammation and immune responses [16]. Se deficiency has been associated with viral and bacterial infections, both in model systems and clinical studies [17,18]. The spread and mutation rate of influenza and Coxsackie virus were highly elevated in Se-deficient animals [19,20,21]. Accordingly, Coxsackie virus infection causing Keshan disease, i.e., a severe congestive cardiomyopathy, develops endemically in Se-deficient populations [10]. The biologically meaningful threshold currently used to define Se deficiency and sufficiency, respectively, has been based on the occurrence and prevention of this endemic infectious disease [22], or on the full expression of selenoproteins [23]. The strong interrelations between virus biology and infections, nutrition and human health are supported by recent analyses of patients with COVID-19, where a positive association of cure rate with regional Se status was observed in China [24], along with an increased mortality risk by Se deficiency in European patients [25].

There are also a number of studies and reviews highlighting a potentially important role of Zn supply, Zn status and Zn distribution in COVID-19, suggesting supplemental Zn as a promising therapeutic adjuvant [26]. Virus replication is enhanced in Zn deficiency, and supplemental Zn can inhibit virus spread and proliferation, as shown in preclinical studies and infected subjects [26,27,28]. It is assumed that Zn deficiency predisposes to severe COVID-19 [29], and accordingly high death rates of COVID-19 patients with Zn deficits have been observed in clinics [30,31].

As Belgium has been heavily affected by COVID-19, we studied the trace element status of a set of consecutive patients admitted to two hospitals in the city of Ghent. We investigated the possible interrelationship between COVID-19 severity and mortality risk with the trace element status and the prevalent comorbidities diabetes mellitus, obesity, chronic cardiac disease and cancer.

## 2. Materials and Methods

### 2.1. Study Design and Participants

A cross-sectional study of patients with COVID-19 was conducted at Ghent University Hospital (UZ Gent) and AZ Jan Palfijn Hospital in Ghent (JPH Ghent), Belgium. The study was conducted in accordance with the Declaration of Helsinki. Ethical counselling was provided by the local Ethics Committee of JPH Ghent and UZ Gent, and approval was granted (BC-07492). All patients enrolled or next of kin provided written informed consent.

Total trace elements (Se, Zn, Fe and Cu) along with GPX3 activity and selenoprotein P (SELENOP) levels were determined from serum (JPH Ghent) or plasma (UZ Gent) of the patients, essentially as described [25]. The group of patients consisted of subjects with proven SARS-CoV-2 infection. The observational study was conducted over a predefined time span with slightly different study protocols at the two study sites, i.e., UZ Gent (study 1) and JPH Ghent (study 2). The inclusion criteria comprised adult age (18–100 years) and a positive COVID-19 diagnosis, as based on detection of SARS-CoV-2 viral RNA using routine RT-PCR analysis as described [27]. The full study cohort consisted of *n* = 138 patients, 79 of whom enrolled into study 1 and 59 into study 2. Five patients from study 1 were transferred to another facility during the study, and one patient was still hospitalized at the end of the study period. These patients were excluded from the clinical analyses. About half of the COVID patients (52%) participating in both studies were aged above 65 years, the usual retirement age in Belgium, and 17% were above 80 years old. Information on age, COVID-19 diagnosis and sex was available for both study 1 and study 2. Data on potential risk factors for severe COVID-19, such as information on diabetes mellitus, malignant neoplasm, obesity and chronic cardiac disease, were available in the database of study 1 only. This information along with age, gender distribution and BMI is presented in Table 1.

Blood plasma (study 1, UZ Gent) or serum (study 2, JPH Gent) was collected from the patients at day one of hospital admission (T1). In study 1, an additional sample was drawn 7 days later (T2), and at discharge or during an outpatient consultation (T3) in case the patient had already been discharged. In study 2, samples were collected solely on day one (T1) of admission to the hospital. The serum or plasma samples were aliquoted, stored at −80 °C, and one aliquot was sent on dry ice for analysis of trace elements and additional biomarkers to the analytical lab in Berlin (Charité–Universitätsmedizin Berlin, Germany). All measurements were conducted by technicians and scientists blinded to the clinical information. Reference values were derived from a large dataset of adult subjects participating in the European Prospective Investigation into Cancer and Nutrition (EPIC) study, analysed by the same technology [30,32].

### 2.2. Assessment of Disease Severity

Disease severity was assessed by the treating physicians during the hospital stay at both study sites separately, by slightly different parameters, which were harmonized to a joint classification scheme:

1. Class A (mild): no infiltrates on chest X-ray or CT thorax (study 1), or asymptomatic disease severity as assessed by the treating physician (study 2)
2. Class B (moderate): fever or history of fever or respiratory symptoms (cough, cough with sputum, wheezing, chest pain, shortness of breath, lower chest wall indrawing) and infiltrates on chest X-ray or CT thorax (study 1), or symptoms of infection, little (<3 L) oxygen requirement and fast discharge (study 2)
3. Class C (severe): respiration rate >30/min or SpO2 <93% (rest) or PF-ratio <300 mmHg at any time during hospitalization (study 1), or moderate disease severity with oxygen deficiency (study 2)
4. Class D (critical): mechanical ventilation or septic shock or ICU stay at any time during hospitalization (study 1), or severe oxygen deficiency (or intubation), difficult recovery, signs of severe infection (study 2)
5. Class E (death): disease severity finally resulting in death (study 1, study 2).

As class A with only mild (asymptomatic) disease severity contained only 15 subjects, classes A and B were merged into a new class of patients (class A + B), all having low oxygen requirements, for part of the data processing. These patients experienced a mild to moderate disease severity. The number of patients classified in the different disease severity classes as function of comorbidities, age class and gender is presented in Table 1.

### 2.3. Analysis of Blood Samples

Serum and plasma samples, respectively, were analysed at Charité–Universitätsmedizin Berlin, essentially as described earlier [25,30,32]. Total reflection X-ray fluorescence (TXRF) was used to determine the total serum or plasma concentrations of Cu, Fe, Se, and Zn using a benchtop TXRF spectrometer (S4 T-STAR, Bruker Nano GmbH, Berlin, Germany). Samples were diluted with a gallium standard, applied to polished quartz glass slides and dried overnight. Seronorm serum standard (Sero AS, Billingstad, Norway) served as control in each analytical run. The concentrations measured were within the specified range of the standard, and the inter-assay coefficient of variation (CV) was below 5% at a concentration of 45 µg Se L^−1^ serum or plasma. SELENOP concentrations were measured by a validated sandwich ELISA method according to the manufacturer’s instructions (selenOmed GmbH, Berlin, Germany). Quality of measurements was verified by including two serum standards in each assay run. The inter-assay CV was below 12% during the analyses. The activity of extracellular GPX3 was assessed by a coupled enzymatic test procedure monitoring nicotinamide adenine dinucleotide phosphate (NADPH) consumption at 340 nm. Briefly, serum or plasma samples were incubated with enzyme buffer containing 3.4 mM reduced glutathione (GSH), 0.27 mg mL^−1^ NADPH, 1 mM NaN_3_, and 300 mU/mL glutathione reductase. The enzymatic reaction was started by hydrogen peroxide, and consumption of NADPH was monitored at 340 nm. Inter- and intra-assay CVs were below 15%.

### 2.4. Statistical Analysis

Data were analyzed using IBM SPSS Statistics (Version 27, New York, NY, USA) or GraphPad Prism (GraphPad Software Inc., version 9, San Diego, CA, USA). Normality of data was assessed by a Shapiro Wilk test. Zn, Se and GPx data were normally distributed, and a Student *t*-test was used to compare groups. As SELENOP, Fe and Cu data were not normally distributed, the non-parametric Mann–Whitney U test was used to compare groups. Significances of differences in mortality between Zn and Se deficient and sufficient subgroups and subgroups with and without comorbidities were assessed using the chi-square likelihood ratio method. All statistical tests were two-sided, and *p*-values < 0.05 were considered significant; * *p* < 0.05, ** *p* < 0.01, and *** *p* < 0.001. As this is an exploratory analysis, *p*-values should be interpreted descriptively, and no adjustment for multiple testing was adopted.

## 3. Results

### 3.1. Trace Element Status at Hospital Admission

All of the patients of study 1 were analysed at hospital admission (T1); Cu, Fe and Zn status were determined as total element concentrations in plasma, whereas Se status was assessed by three interrelated biomarkers, i.e., total Se concentration, SELENOP levels and GPX3 activities. The data are separated into subgroups of male and female patients, patients above and below 65 years old, and patients with or without malignant neoplasm, diabetes, chronic cardiac disease and obesity, respectively. The results indicate that Cu levels were adequate in the majority of samples. Fe levels were higher in male patients, whereas Cu levels were higher in female patients. Plasma GPX3 activities were higher in patients below 65 years old. The trace element concentrations were not significantly different in relation to diabetes, chronic cardiac disease or obesity, but tumour patients presented with significant deficits in Zn and SELENOP (Table 2). Out of the 10 subjects with the lowest plasma Zn levels among the entire cohort, seven were from the small group of cancer patients. Three of the five patients with the lowest blood Fe concentrations died during the study; these non-survivors also displayed profound simultaneous Se and Zn deficits, and were thus exhibiting a kind of universal trace element-deficiency.

Criteria for an insufficient Se status have been established for serum or plasma Se [33], i.e., total Se < 45.7 µg L^−1^, and for SELENOP [25,32], i.e., SELENOP < 2.56 mg L^−1^. Applying these thresholds, a severe Se deficit was detected in 24.6% (based on total Se), and 72.5% (based on SELENOP) of the patients, respectively. In comparison to healthy European adults [32], the patients displayed strongly depressed total Se (mean ± SD; 59.2 ± 20.6 vs. 84.4 ± 23.4 µg L^−1^) and SELENOP (mean ± SD: 2.2 ± 1.9 vs. 4.3 ± 1.0 mg L^−1^). Three patients only (1.8%) had Se concentrations above 120 µg L^−1^, which is required for saturated SELENOP expression [34]. Two patients displayed a most severe Se deficit with <12 µg L^−1^ only, i.e., a condition hardly ever observed before in previous clinical cohorts analysed at the Berlin site.

### 3.2. Relation between Trace Element Status and Disease Severity

**Figure 1:**
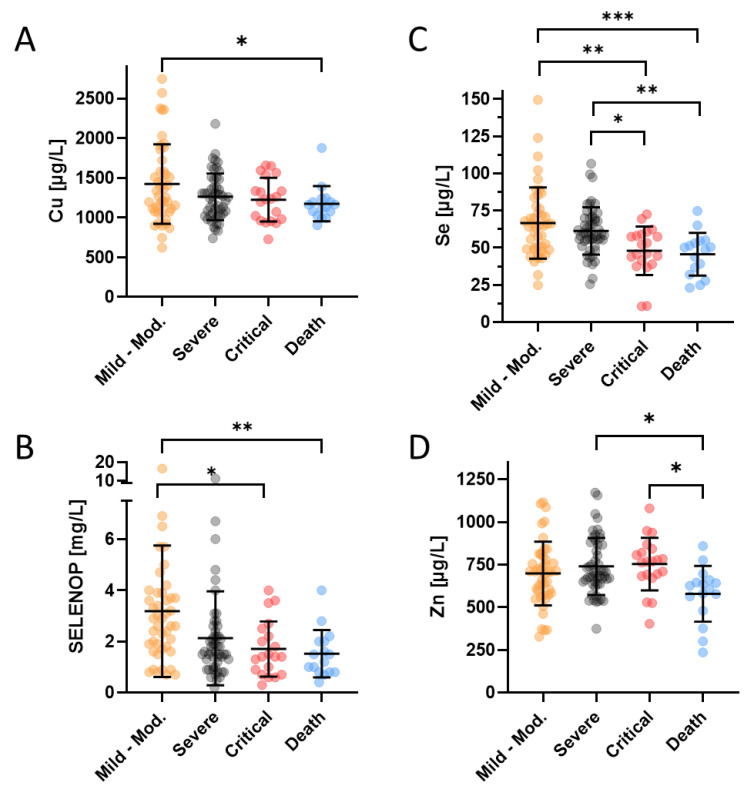
Trace element and SELENOP concentrations of COVID-19 patients in relation to disease severity (not including the six COVID-19 patients transferred to another facility during the study or still hospitalized at the end of the study). The classification of disease severity ranged from (very) mild to moderate (class A and B, Mild-Mod.), over severe (Severe) and critical (Critical) to death (Death). (**A**) The Cu status was depressed in the non-survivors only. (**B**) Se as well as (**C**) SELENOP concentrations decreased with disease severity, frequently falling below the thresholds for severe deficiency of <45 µg/L (Se) and <2.56 mg/L (SELENOP), respectively. (**D**) Zn status was similarly depressed as the Cu status in non-survivors only, dropping below the threshold for severe Zn deficiency (<660 µg/L) in the majority on non-surviving patients. Total number of patients; *n* = 132. The comparison was conducted by a non-parametric Mann–Whitney U test, * *p* < 0.05, ** *p* < 0.01, *** *p* < 0.001.

Disease severity of all the patients enrolled into the study was assessed by the treating physicians and categorized into five different classes i.e., class A (mild), class B (moderate), class C (severe), class D (critical), and class E (death), respectively. For the statistical analysis of similarly-sized groups, patients in the two lowest severity classes A and B were combined. There was a slightly reduced Cu status in patients who finally succumbed to the infection in comparison to survivors with mild to moderate disease (Figure 1A). The Se and SELENOP status showed consistent and relatively linear downward trends with disease severity (Figure 1B,C). Similar to Cu, the Zn levels were particularly depressed in the group of non-survivors, but not different between the groups of mildly, moderately or severely diseased patients (Figure 1D).

All of the COVID-19 patients who finally died exhibited Se deficiency already at hospital admission (T1). The Se concentrations in this group ranged from as low as 23 µg L^−1^ to 64 µg L^−1^, i.e., frequently in the range of severe Se deficits (<45 µg L^−1^) [33], and always below the threshold for moderate Se deficiency (<70 µg L^−1^) [35]. The patient of the entire population with the lowest Se concentration was a woman of 48 years of age, who was diagnosed with diabetes and obesity. She went through a very critical disease course and resided in the hospital for 32 days, 15 of which were on the ICU. Another young 28-year old female patient with diabetes, obesity and hypothyroidism had the second lowest Se level of the whole cohort (10.9 µg L^−1^), and an extremely low SELENOP concentration (0.3 mg L^−1^). She also went through a very critical disease course and stayed in hospital for 36 days, 22 of which on the ICU. In comparison, the patients who experienced a mild to moderate disease course (class A + B, *n* = 45) displayed relatively preserved SELENOP status (average ± SD; 3.2 ± 2.6 mg L^−1^) as compared to the critically diseased or non-survivors (class C–E, *n* = 87, average ± SD; 1.9 ± 1.6 mg L^−1^).

### 3.3. Clinical Relevance of Se and Zn Deficits for Disease Severity

**Figure 2:**
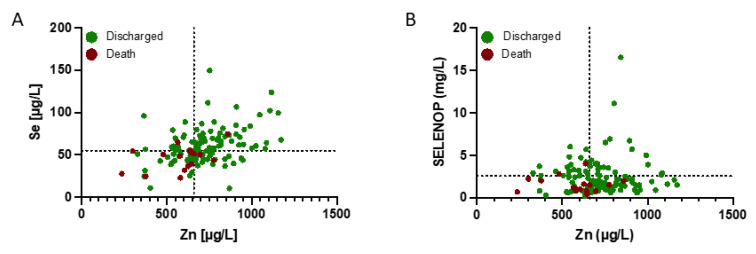
Zinc, Se and SELENOP status (plasma or serum concentration) of COVID-19 patients in relation to survival. The patients were separated into survivors (green dots) and non-survivors (dark red dots). A high fraction of the non-survivors displayed a combined deficiency in (**A**) Se and Zn, and (**B**) SELENOP and Zn concentrations at admission to hospital, in comparison to the survivors. Thresholds for deficiency are indicated by the broken lines.

The data on trace element status were analysed in relation to survival. Many of the patients displayed a combined Se and Zn (Figure 2A) and/or SELENOP and Zn deficit (Figure 2B). The majority of non-survivors (70.6%) was characterized by Se below 55.2 µg L^−1^ and Zn below 660 µg L^−1^ at admission (T1). These concentrations are defined as Se and Zn deficiency thresholds and indicated by broken lines in Figure 2.

### 3.4. Interrelation between Trace Element Status and Mortality in Relation to Comorbidities

Next, the relevance of Se and Zn deficits was assessed for the mortality risk of COVID-19 patients with comorbidities (Table 2). As expected, younger age was positively associated with survival, and two fatalities only were observed in the group of patients until 65 years of age (mortality rate: 4.4%), whereas eight patients died in the group of patients above 65 years of age (mortality rate: 28.6%).

Besides senior age, mortality risk was strongly associated with Zn and Se deficiency. Age has a clear impact on death risk in the Se deficient subpopulation and an almost significant impact in the Zn deficient subpopulation. In the Zn and Se sufficient subpopulation, this effect of age on mortality was not observed. Relations between Zn and Se status and mortality were significant in the senior patient group above 65 years of age, but not in the younger patient group. Both Se and Zn status can thus be considered as an appropriate predictor for survival mainly in the senior patient group. Not a single patient with a sufficient Se status succumbed to COVID-19 in the senior patient group above 65 years of age, whereas all non-survivors displayed a strong Se deficit ([Se] < 55.2 µg L^−1^) already at hospital admission. For the diabetic patients, low Se and Zn status at admission were particularly associated with high mortality risk. Whereas Zn and Se deficiency are both associated with an elevated death risk in the subgroup of diabetic patients, this relation was not significant for Zn in the subgroup not suffering from diabetes. Therefore, and because the mortality is very high in the Zn deficient subgroup of diabetic patients (50.0%), particularly Zn status is considered as a suitable predictor for the death risk of patients suffering from diabetes. Patients with malignant neoplasm and chronic cardiac disease displayed a significant difference in mortality between the subgroups having a deficient and sufficient Se status, whereas such differences were not observed for Zn sufficient and deficient subpopulations. Selenium status can thus be considered as the most appropriate predictor for survival in the subgroups of patients with malignant neoplasm and chronic cardiac disease. In the population of obese patients, not a single patient having a sufficient Se or Zn status succumbed to COVID-19. The association between micronutrient status and death risk was particularly significant for Zn.

In both the Se and Zn deficient subpopulations and in the Se and Zn sufficient subpopulations, all of the comorbidities discussed above do not have a significant impact on death risk. This indicates that a low Se and Zn status of the patient, which may also be attributed to the occurrence of a comorbidity in some cases, rather than the presence of the comorbidity itself results in a higher death risk. A combined Se and Zn deficit may thus constitute a most relevant risk factor for a fatal COVID-19 outcome, and the status of Se and Zn may modify mortality risk from COVID-19 differentially in patients of different age classes and patients with chronic cardiac disease, obesity, diabetes or cancer (Table 3).

The relevance of a sufficiently preserved Se status at admission for surviving the infection becomes most obvious when considering the full cohort of patients, i.e., combining study 1 and 2. Here, the younger patients (until 65 y) who experienced a critical disease course and/or died (class D + E, *n* = 14) displayed a significantly lower Se status as compared to the less severely affected patients (classes A–C, *n* = 58) (46.7 ± 17.8 µg L^−1^ vs. 63.1 ± 18.3 µg L^−1^). Similarly, the SELENOP status was particularly depressed in the group of younger COVID-19 patients (until 65 y), who went through a critical disease course and/or died (classes D + E) as compared to those with a mild to moderate (classes A + B) disease course (1.4 ± 0.6 mg L^−1^ vs. 3.1 ± 3.1 mg L^−1^). The SELENOP status in these critically diseased or non-surviving younger patients was also low in comparison to critically diseased or non-surviving senior patients (>65 y: 1.8 ± 1.2 mg L^−1^), or to less severely affected seniors (3.0 ± 2.1 mg L^−1^). Of the 13 COVID-19 patients with malignant neoplasm in study 1, seven were below and six were above 65 years of age, excluding age as the dominant factor for Se deficiency in the cancer patients.

### 3.5. Trace Element Dynamics from Hospital Admission to Discharge

**Figure 3:**
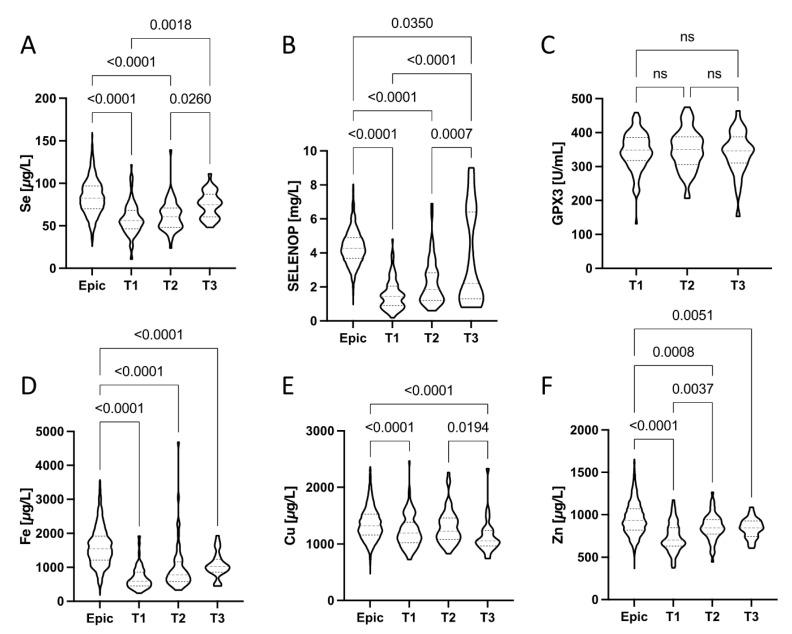
Dynamic changes during disease in (**A**) Se, (**B**) SELENOP, (**C**) GPX3, (**D**) Fe, (**E**) Cu, and (**F**) Zn status (in plasma) of the hospitalized COVID-19 patients in comparison to healthy European adults. Plasma samples were available at the time of hospital admission (T1), during treatment (T2) and during or after discharge (T3). Violin plots corrected for outliers are presented. All data sets were compared by non-parametric tests; *p*-values are indicated above the respective groups of values.

The trace element dynamics in plasma from the patients enrolled into study 1 (UZ Gent) was analysed at three relevant time points, i.e., at admission to hospital (T1), during treatment and hospitalisation (T2), and during or after discharge (T3). The depressed Se status at T1, reflected in relatively low total Se and SELENOP concentrations, progressively improved in the majority of patients towards discharge (Figure 3A,B). None of the patients was still presenting a Se status below 55.2 µg L^−1^ at T3 (Figure 3A). However, 88% of the COVID-19 patients still had an inadequate Se status based on the Se requirement for optimization of GPx3 activity (i.e., [Se] > 90 µg L^−1^) at that time. Among the surviving COVID-19 patients with severe Se deficit at admission (T1), who were also sampled at T3, none of the four had reached an adequate Se status ([Se] > 90 µg L^−1^). The two surviving COVID-19 patients who had an adequate Se status at T3 ([Se] > 90 µg L^−1^) had been already above this threshold at admission (T1). A majority of patients (56%) still had a deficient SELENOP status ([SELENOP] < 2.56 mg L^−1^) at T3, indicating a lack of sufficient Se supply or an ongoing compromised hepatic SELENOP biosynthesis rate during disease and recovery (Figure 3B). Plasma GPX3 activities were not significantly different at the consecutive time points of analysis and showed little variations only (Figure 3C). Total plasma Fe concentrations (Figure 3D) showed strong differences between the healthy subjects (Epic) and the patients, irrespective of time point analyzed. There was an apparent tendency of recovering Fe concentrations in the patients, albeit without reaching statistical significance. Plasma Cu status appeared slightly depressed in the patients as compared to healthy controls (Epic), but still within the reference range in the majority of samples (Figure 3E). Total plasma Zn (Figure 3E) was strongly depressed in particular at the time of admission (T1), recovered relatively fast, and the majority of patients displayed no obvious Zn deficit during or after hospital discharge at T3.

## 4. Discussion

Infections with SARS-CoV-2 cause mild or severe COVID-19 course, and predictive markers for a better stratification of patients at hospital admission, are needed. In this study, we report on particularly low total Se, total Zn and depressed SELENOP concentrations in the majority of patients who were hospitalized with proven COVID-19 infection at one of two hospital sites in Ghent, Belgium. The analysis of patients with comorbidities highlighted a particularly pronounced deficit of plasma Se, Zn, Fe and SELENOP in cancer patients, along with a strong positive interrelation of preserved Se status with high survival chances under these conditions. Similarly, disease severity and length of hospital stay were associated with Se and SELENOP deficits, and already detectable at admission. The focused analysis of trace elements at admission in relation to mortality rate highlighted Zn deficiency as a particular relevant risk factor for patients with diabetes mellitus. The majority of non-survivors were characterized by a combined Se and Zn deficit, and both trace elements recovered in blood during hospital stay and towards discharge as indicators of high survival chances.

The findings are in general agreement with prior reports on trace elements in COVID-19, and their interrelation with mortality risk. A seminal publication from China indicated higher cure rates from COVID-19 in areas with better habitual Se status [24]. This interrelation was also observed for individual patients, where low and declining Se concentrations in blood were associated with poor survival odds [25]. Notably, an increased mortality risk with Se deficiency was not restricted to the elderly population, but also observed in relatively young and otherwise healthy patients without known comorbidities in a respective study conducted in India [36]. The depressed Se status is likely caused directly by the viral infection, as the concentrations observed in patients with COVID-19 are far below reference ranges and are recovering towards normal values in the majority of survivors. In terms of how far low Se predisposes to infection, severe disease course and hospitalization is currently unknown, as the samples analysed at hospital admission are not representative of the full population of infected subjects. Still, a compromised immune response to SARS-CoV-2 infection and suppressed immune cell activity under Se deficiency can be assumed and would be in line with preclinical studies and circumstantial evidence from other infections [37,38]. The negative acute phase response of serum SELENOP and Se might close a vicious cycle of declining Se status with ongoing inflammation, unless counteracted by anti-inflammatory medication, successful immune response or sufficiently high Se supply after infection and during hospital stay [18,39,40,41,42]. The interrelationship of viral or bacterial infection, activated immune response, and declining Se status does not constitute a unique characteristic of COVID-19, but was similarly observed before in other diseases, like sepsis, HIV, influenza or coxsackievirus infection [20,43,44,45,46]. Similarly, an association of low Se status with high mortality risk is a common finding in studies with severely diseased patients, in particular with patients requiring support on the intensive care units [43,44,47].

Besides Se, Zn constitutes a second most relevant trace element for immune cells, inflammation and for combating SARS-CoV-2 infections [13,48,49]. Deficiency in Zn has been described as a risk factor for mortality in COVID-19, and plasma Zn concentrations at admission were associated with 21% and 5% mortality, respectively, in relation to a plasma Zn threshold of 500 μg L^−1^ [31]. Accordingly, no loss to COVID-19 was observed in an independent clinical study, when the patients displayed a replete Zn status of ≥800 μg L^−1^ at admission to hospital, in contrast to an 18.5% mortality rate below this threshold [50]. However, low Zn status seems to recover relatively fast after hospitalization, as reported before [30], and as observed in the current study (Figure 3F). The transient decline may reflect a dynamic redistribution between blood and immune cells.

In view of the strongly elevated mortality observed in the group of patients with Se concentrations at hospital admission below 55.2 µg L^-1^, the current threshold for defining deficiency (>0.25 µM, equal to serum or plasma Se of 20 µg L^−1^), which was based on the prevention of cardiomyopathy upon infection by the Coxsackie virus, should be reconsidered [51]. Besides COVID-19 mortality, low Se status predisposes to a number of diseases, including cancer, cardiovascular disease or autoimmune thyroid disease [52]. Together with the growing body of evidence linking Se deficit to increased mortality risk in COVID-19, it appears timely to raise the threshold and consider supportive nutrition in hospital and ideally avoid such low supply in the general population, as has successfully been done in Finland for more than three decades [53]. A cross-European analysis of COVID-19 mortality rates in the different countries in relation to their habitual Se intake and blood Se status would be most suitable to test this notion and consider appropriate preventive measures.

The mortality in our study was elevated in Se and Zn deficiency, in particular within the groups of obese, chronic cardiac disease, cancer and diabetes patients. These conditions confer an increased mortality risk in COVID-19, and are often associated with (sub-)clinical inflammation, again potentially closing a vicious self-amplifying cycle [54,55]. As cancer and chronic cardiac disease were associated with a higher mortality particularly in the Se-deficient patients, obesity was associated with a higher mortality in Zn-deficient patients and both Se- and Zn-deficiency conferred increased mortality risk in particular for diabetes patients, it is hypothesized that Se and Zn deficiency may aggravate the negative impact of cancer, obesity, chronic cardiac disease and diabetes on an adequate immune response in COVID-19.

Both the Se and Zn status, in particular SELENOP, tended to improve during recovery. Furthermore, the younger patients who had to go through a critical disease course or even lost their life during the hospital stay often exhibited pronounced Se deficiencies at admission, which were even more severe compared to older patients going through a similar disease severity. From these observations, together with the relationship between low Se status, disease severity and mortality risk, we hypothesize that a sufficiently high Se status is a most critical factor for COVID-19 course in young subjects, and in patients with comorbidities. In order to translate these findings into a useful diagnostic scheme for assessing and predicting COVID-19 course at hospital admission, the following scheme has been developed and will be tested in follow-up studies:

Newly admitted patients with proven or suspected COVID-19 will be assessed by a trace element-related severity score (TERS), whereby the information on laboratory assessed Se, SELENOP and Zn concentrations are combined for each patient and categorized by a TERS of 0 to 3. TERS represents the sum of X + Y + Z, with:

X = 1 if [Zn] > 660 µg L^−1^ (otherwise X = 0)

Y = 1 if [Se] > 55.2 µg L^−1^ (otherwise Y = 0)

Z = 1 if [SELENOP] > 2.0 mg L^−1^ (otherwise Z = 0)

Applying the TERS system to our full study cohort, meaningful interrelations are observed (Table 4).

In the age group of senior patients above 65 years of age, the predictive accuracy of this model seems helpful to identifying patients with high risk for mortality, and inversely to identify those ones who will likely develop a mild disease course only. Over three quarters (78%) of the senior COVID-19 patients with TERS = 0 succumbed to the infection, whereas the majority (79%) of the senior patients who survived and displayed mild disease to moderate symptoms only had a TERS = 2 or 3. Only 5% of the senior patients going through a mild to moderate disease course were found in the group with a TERS of 0, and no senior patient who died had a score of 2 or 3 at admission to the hospital. The same applies to the full patient group, where one patient only with a TERS of 3 did not survive, in contrast to all others with a TERS of 2 or 3 who survived COVID-19, underlining the potential value of this scoring system.

The assessment of COVID-19 severity by TERS may help the treating physicians to take appropriate decisions for treatment of the patients, for considering trace element supplements and probably for supporting in particular the patients with comorbidities who are at particular risk for Se and Zn deficiency and an impaired metabolism and uptake of these two essential trace elements, in association with higher mortality risks. A discrepancy between the clinical severity assessment and TERS may help to identify patients with particular requirements.

Among the particular strengths of our study are the consequent assessment of a set of important trace elements directly at hospital admission and of some longitudinal observations of trace element dynamics during the course of COVID-19 in relation to age and comorbidities of patients belonging to different age groups. The analyses remote from the clinical sites by personnel blinded to the clinical phenotype using validated techniques constitutes another strength. Among the limitations is the purely observational nature of our study, excluding cause-effect insights into the reasons for trace element deficiencies, the lack of data on cytokines and the fact that different criteria were used by the physicians in both participating hospitals to assess disease severity, and the limited size of our study cohort. Nevertheless, the results obtained and, in particular, the new TERS system developed for identifying patients at particular risk of a severe COVID-19 course may be of help for clinical decisions on treatment and adjuvant micronutrient supply.

## 5. Conclusions

Our data confirm that an insufficient Se (total Se and SELENOP) and Zn status at admission to the hospital is associated with an exceptionally high mortality risk and severe disease course with COVID-19. Our study contributes to the set of informative biomarkers in COVID-19, with potential relevance also to other similar infectious diseases. In view of the predictive accuracy of Se and Zn deficiency as mortality risk factor at hospital admission, supplemental Se and Zn supply should be considered to support the immune system, in particular for patients with inflammation-related comorbidities like cancer or diabetes mellitus. However, causality remains unknown due to the observational nature of this study. Randomized clinical trials are needed to test whether Zn and Se supplementation to patients with diagnosed deficiencies are relevant for reducing mortality risk, decreasing hospitalization time, accelerating recovery and potentially even preventing post-COVID-19 syndrome. From our point of view, there are no obvious reasons against such supportive adjuvant measures, as long as clinically recommended dosages are not surpassed.

## Figures and Tables

**Table 1:** Age and gender distribution of the population of study 1 (UZ Gent), occurrence of comorbidities in different subpopulations, and body mass index (BMI) and number of patients in each disease severity class as function of comorbidities, gender and age for study 1.

		Cancer	Diabetes	Obesity	Cardiac Disease	BMI	Disease Severity A/B/C/D/E *
	*n*	*n*	*n*	*n*	*n*	Mean ± SD	*n*
All patients	79	13	24	22	22	28.5 ± 5.2	2/8/39/20/10
Male Female	55 24	9 4	15 9	16 6	19 3	28.9 ± 5.2 27.0 ± 5.1	1/7/24/15/8 1/1/15/5/2
Malignant neoplasm+	13	13	2	3	5	27.6 ± 4.5	0/1/5/4/3
Malignant neoplasm−	66	0	22	19	17	28.7 ± 5.4	2/7/34/16/7
Diabetes+	24	2	24	14	9	30.2 ± 5.9	0/2/11/7/4
Diabetes−	55	11	0	8	13	27.7 ± 4.8	2/6/28/13/6
Obesity+	22	3	14	22	7	34.5 ± 4.1	0/3/8/9/2
Obesity−	48	8	9	0	15	25.8 ± 3.3	1/4/26/10/7
Chronic cardiac disease+	22	5	9	7	22	27.7 ± 5.5	2/2/7/6/5
Chronic cardiac disease−	57	8	15	15	0	28.8 ± 5.1	0/6/32/14/5
Below 65 years	46	7	11	14	7	29.9 ± 7.4	0/7/24/13/2
65–80 years	26	4	8	5	9	26.6 ± 4.7	1/1/15/6/3
Above 80 years	7	2	5	3	6	26.8 ± 3.0	1/0/0/1/5

* Disease severity: A: mild, B: moderate, C: severe, D: critical, E: death; *n*: values per group; SD: standard deviation; malignant neoplasm+, diabetes+, chronic cardiac disease+ and obesity+: clinically diagnosed cancer, diabetes mellitus, chronic cardiac disease and obesity, respectively.

**Table 2:** Trace element status at hospital admission.

		Se [µg L^−1^]		Zn [µg L^−1^]		SELENOP [mg L^−1^]		Cu [µg L^−1^]		Fe [µg L^−1^]		GPX3 [U L^−1^]	
	*n*	Mean	SD	Mean	SD	Mean	SD	Mean	SD	Mean	SD	Mean	SD
All patients of study 1	79	56.6	16.9	735	166	1.6	0.9	1168	229	689	331	349	51
Male	55	57.6	16.1	729	164	1.6	1.0	1125	190	737	352	351	54
Female	24	54.2	18.7	748	173	1.5	0.9	1268	279	578	252	346	45
*p*-value		0.405		0.645		0.924		**0.018**		**0.038**		0.726	
Until 65 years	49	58.2	19.1	747	182	1.5	0.8	1170	245	703	367	360	50
Above 65 years	30	53.9	12.4	715	135	1.7	1.1	1164	205	666	269	332	48
*p*-value		0.275		0.409		0.808		0.856		0.840		**0.017**	
Malignant neoplasm+	13	50.2	9.1	623	113	1.1	0.6	1100	186	560	187	336	52
Malignant neoplasm−	66	57.8	17.9	757	166	1.7	0.9	1181	235	714	348	352	51
*p*-value		0.140		**0.007**		**0.022**		0.200		0.202		0.315	
Diabetes+	24	52.2	15.5	767	171	1.4	0.8	1156	191	634	259	340	42
Diabetes−	55	58.5	17.3	720	163	1.6	1.0	1173	245	713	358	353	54
*p*-value		0.128		0.254		0.343		0.798		0.449		0.279	
Obesity+	22	52.2	16.7	7701	167	1.5	1.0	1199	208	584	209	342	44
Obesity−	48	58.2	16.9	754	165	1.6	0.9	1169	242	741	351	352	52
*p*-value		0.173		0.220		0.383		0.433		0.077		0.425	
Chronic cardiac disease+	22	55.5	15.0	737	127	1.3	0.9	1137	217	722	365	322	49
Chronic cardiac disease−	57	57.0	17.7	734	180	1.7	0.9	1180	234	676	320	356	51
*p*-value		0.731		0.928		0.134		0.347		0.687		0.064	

Data (plasma concentrations) of all enrolled participants from study 1 (UZ Gent); *n*: values per group; SD: standard deviation, *p*-value: significantly different between the subgroups; significant differences indicated in bold (*p* < 0.05); malignant neoplasm+, diabetes+, chronic cardiac disease+ and obesity+: clinically diagnosed cancer, diabetes mellitus, chronic cardiac disease and obesity, respectively.

**Table 3:** Se and Zn status in relation to age and comorbidities for surviving COVID-19, study 1.

Mortality [%]	Full Group	Zn Deficient	Zn Sufficient	*p*-Value	Se Deficient	Se Sufficient	*p*-Value
Total group	13.7 (10/73)	29.2 (7/24)	6.1 (3/49)	**0.009**	29.0 (9/31)	2.4 (1/42)	**<0.001**
Until 65 years	4.4 (2/45)	7.7 (1/13)	3.1 (1/32)	0.164	6.3 (1/16)	3.4 (1/29)	0.328
Above 65 years	28.6 (8/28)	54.5 (6/11)	13.3 (2/17)	**0.045**	53.3 (8/15)	0.0 (0/13)	**<0.001**
*p*-value	**0.004**	**0.009**	0.244		**0.002**	0.386	
Diabetes+	16.7 (4/24)	50.0 (3/6)	5.6 (1/18)	**0.018**	30.8 (4/13)	0.0 (0/11)	**0.018**
Diabetes−	12.2 (6/49)	22.2 (4/18)	6.5 (2/31)	0.111	27.8 (5/18)	3.2 (1/31)	**0.012**
*p*-value	0.611	0.208	0.899		0.857	0.433	
Malignant neoplasm+	25.0 (3/12)	28.6 (2/7)	20.0 (1/5)	0.733	42.9 (3/7)	0.0 (0/5)	**0.047**
Malignant neoplasm−	11.4 (7/61)	29.4 (5/17)	4.5 (2/44)	**0.010**	25.0 (6/24)	2.7 (1/37)	**0.007**
*p*-value	0.245	0.967	0.255		0.612	0.372	
Cardiac disease+	25.0 (5/20)	42.9 (3/7)	15.4 (2/13)	0.183	41.7 (5/12)	0.0 (0/8)	**0.013**
Cardiac disease−	9.4 (5/53)	23.5 (4/17)	2.8 (1/36)	**0.020**	21.1 4/19	2.9 (1/34)	**0.033**
*p*-value	0.100	0.353	0.132		0.222	0.513	
Obesity+	11.1 (2/18)	33.3 (2/6)	0.0 (0/12)	**0.027**	22.2 (2/9)	0.0 (0/9)	0.082
Obesity−	14.9 (7/47)	26.7 (4/15)	9.4 (3/32)	0.134	31.6 (6/19)	3.6 (1/28)	**0.007**
*p*-value	0.687	0.762	0.158		0.604	0.452	

Trace element status was determined at hospital admission; threshold for deficiency is set at [Zn] <660 µg L^−1^, and at [Se] <55.2 µg L^−1^; numbers in brackets: deceased patients in the subgroup/total number of patients in the subgroup; *p*-values represent significances of differences in mortality between groups presented in the rows and columns according to a chi-square likelihood ratio evaluation; significant differences indicated in bold (*p* < 0.05).

**Table 4:** COVID-19 mortality in relation to trace element-related severity score (TERS) and severity class.

Age Group	TERS	Number (% of Total)	Non-Surviving (Class E) *	Critical (Class D) *	Severe (Class C) *	Mild to Moderate (Class A + B) *
entire cohort	0	17 (13%)	8 (50/47)	2 (10/12)	3 (6/18)	4 (9/24)
	1	40 (30%)	7 (44/18)	8 (40/20)	13 (25/33)	12 (27/30)
	2	45 (34%)	0 (0/0)	6 (30/13)	25 (49/56)	14 (31/31)
	3	30 (23%)	1 (6/3)	4 (20/13)	10 (20/33)	15 (33/50)
above 65 y	0	9 (14%)	7 (58/78)	0 (0/0)	1 (5/11)	1 (5/11)
	1	19 (32%)	5 (42/26)	4 (44/21)	7 (37/37)	3 (16/16)
	2	15 (26%)	0 (0/0)	1 (11/7)	7 (37/47)	7 (37/47)
	3	16 (28%)	0 (0/0)	4 (44/25)	4 (21/25)	8 (42/50)

* in brackets: % of total in class/% of total with this score; thresholds Zn 660/Se 55.2/SELENOP 2.0.

## Data Availability

The data presented in this study are available on request from the corresponding author. The data are not publicly available due to privacy regulations.
